# Supplementary material for: Plasmonic nanoparticle amyloid corona for screening Aβ oligomeric aggregate-degrading drugs
Source: Nat Commun. 2021 Jan 27;12:639. doi: 10.1038/s41467-020-20611-4 (PMC7840768; doi:10.1038/s41467-020-20611-4)
Supplement: Supplementary file 1 — Supplementary Information [file 41467_2020_20611_MOESM1_ESM.pdf]

Supplementary information

for

Plasmonic nanoparticle amyloid corona for screening Aβ oligomeric aggregate-degrading drugs

Dongtak Lee[1], Dongsung Park[1,2], Insu Kim[1], Sang Won Lee[1], Wonseok Lee[3], Kyo Seon Hwang[2], Jeong Hoon Lee[4,*], Gyudo Lee[5,*], Dae Sung Yoon[1,*]

[1] School of Biomedical Engineering, Korea University, Seoul 02841, South Korea

[2] Department of Clinical Pharmacology and Therapeutics, College of Medicine, Kyung Hee University, Seoul, 02447, South Korea

[3] Department of Control and Instrumentation Engineering, Korea University, Sejong 30019, South Korea

[4] Department of Electrical Engineering, Kwangwoon University, Seoul 01897, South Korea

[5] Department of Biotechnology and Bioinformatics, Korea University, Sejong 30019, South Korea

*Corresponding authors: D.S.Y. (dsyoon@korea.ac.kr); G.L. (lkd0807@korea.ac.kr); J.H.L. (jhlee@kw.ac.kr)

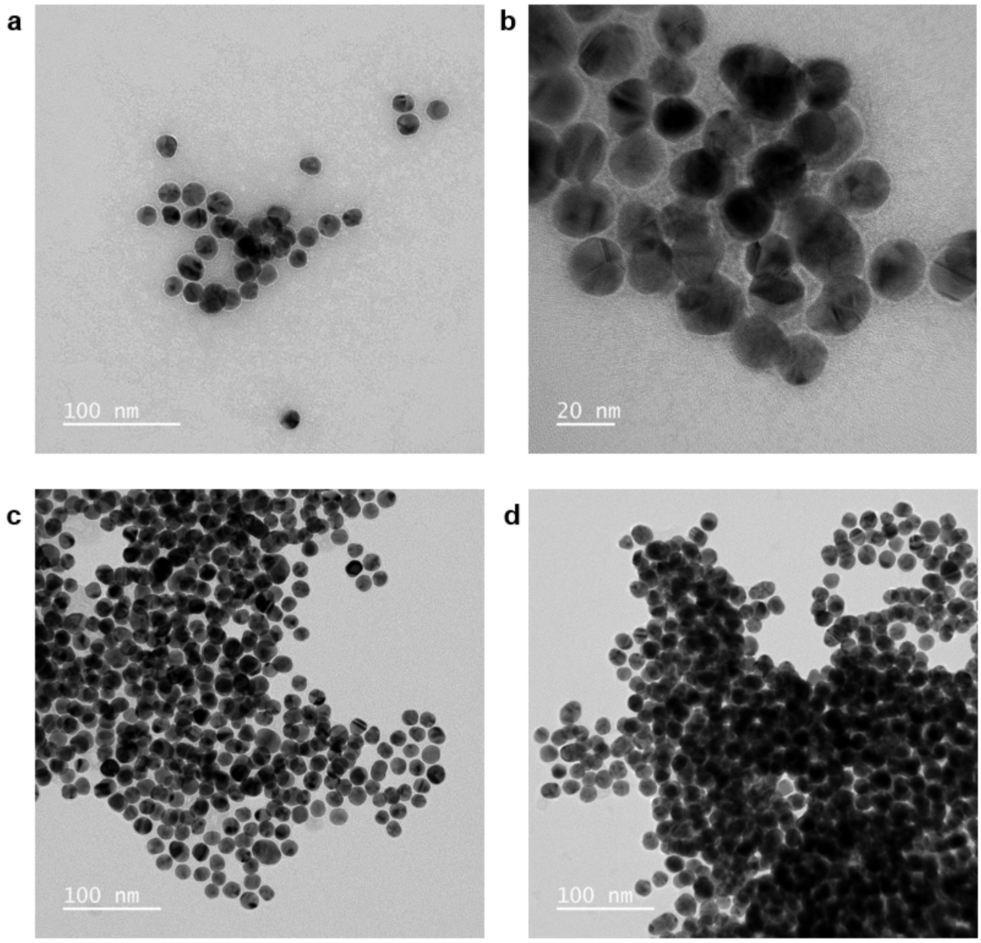

Supplementary Figure 1. HRTEM images of PNACs with Aβ-degrading agents (**a, b**) EPPS (5 mM) or (**c, d**) protease XIV (100 μg mL^{-1}).

The HRTEM images show the aggregation of PNACs treated with Aβ-degrading agents, such as EPPS (5 mM) or protease XIV (100 μg mL^{-1}). Because of the relatively low Aβ degradation ability of EPPS compared to that of protease XIV, the degrees of aggregation of PNACs differ. Specifically, the aggregated area of PNACs treated with EPPS is small (Supplementary Fig. 1a, b), whereas that of PNACs treated with protease XIV is very large (Supplementary Fig. 1c, d). The HRTEM analysis shows that the PNACs are sensitive to differences in the degradation ability of Aβ-degrading agents.

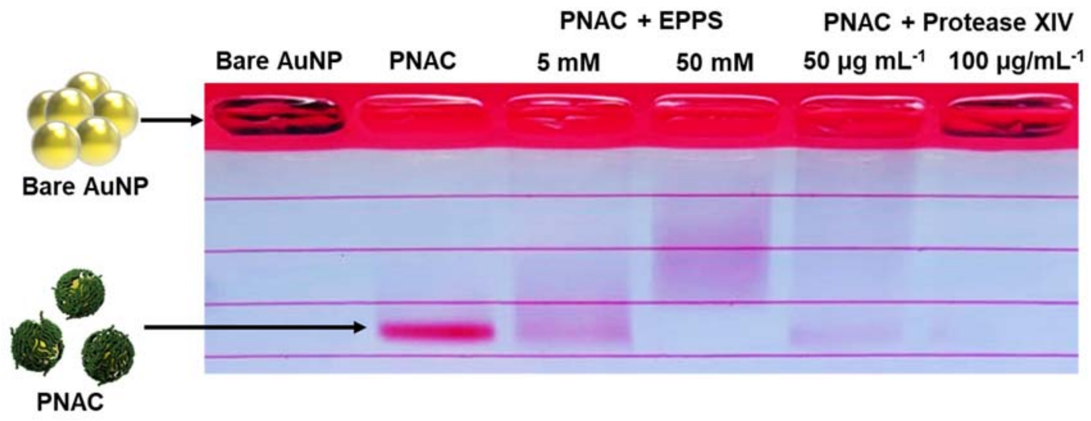

Supplementary Figure 2. Agarose gel electrophoresis of the PNAC samples depending on treatment with different Aβ-degrading agents in 1× Tris-acetate/ethylenediaminetetraacetic acid (TAE) buffer solution. The difference in the color is caused by the degradation of the Aβ oligomeric aggregates on the PNAC surfaces (i.e., optical property changes because of AuNP aggregation).

We performed gel electrophoresis as a suitable assay to ensure the degradation of the oligomeric aggregates on the PNAC surfaces (Supplementary Fig. 2). Before electrophoresis, the bare AuNPs aggregated with each other because of their low stability in the salt conditions provided by the Tris-acetate/ethylenediaminetetraacetic acid (TAE) 1× buffer. Thus, no band is observed. In contrast, the PNAC sample exhibit a distinct band. This is because the PNACs (AuNPs with Aβ oligomeric aggregates) are well dispersed, as the oligomeric aggregates provided good steric stability. With Aβ-degrading agents, such as EPPS and protease XIV, the PNAC band is broadened, indicating that aggregation of the PNACs occurs with the removal of the oligomeric aggregates from the PNACs. The aggregation of the PNACs causes slower movement in the agarose gel, resulting in band broadening.

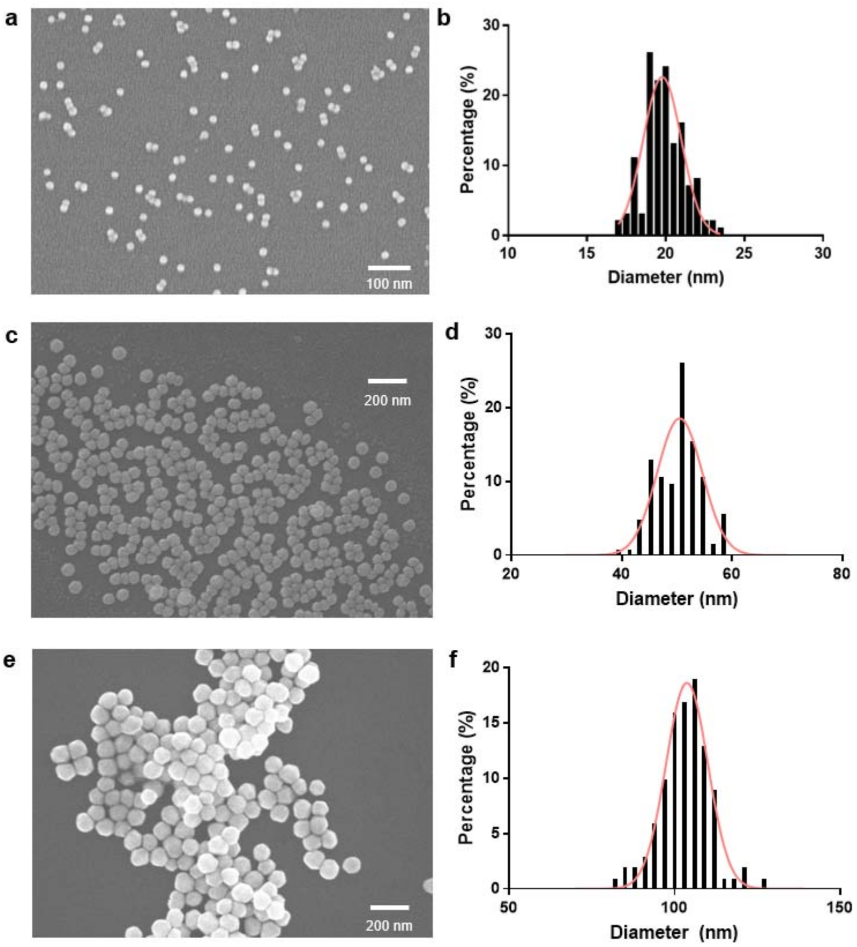

Supplementary Figure 3. **a**, SEM image of 20-nm AuNPs. **b**, Size distribution of 20-nm AuNPs (20.1 ± 1.24 nm, n = 103). **c**, SEM image of 50-nm AuNPs. **d**, Size distribution of 50-nm AuNPs (50.21 ± 4.05 nm, n = 122). **e**, SEM image of 100-nm AuNPs. **f**, Size distribution of 100-nm AuNPs (103.40 ± 7.43 nm, n = 103). The average size of the AuNPs was determined using ImageJ software (NIH, USA).

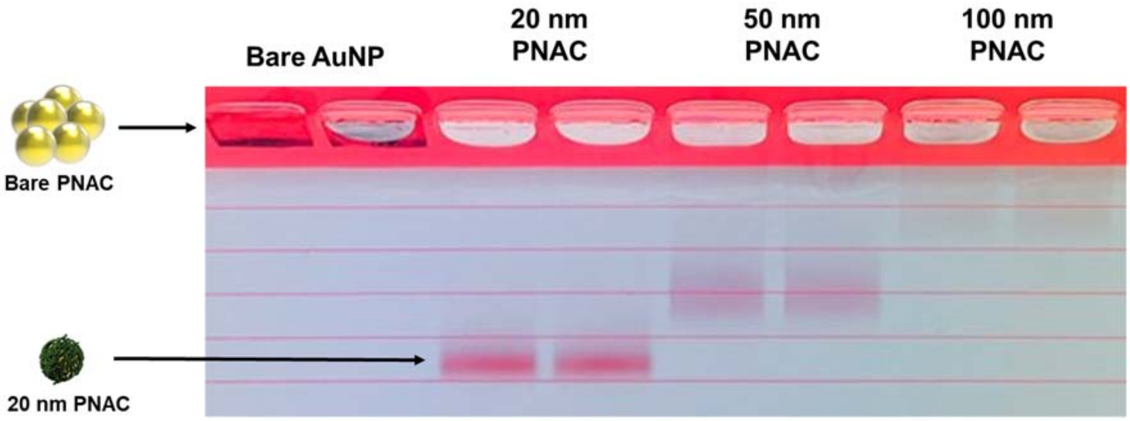

Supplementary Figure 4. Agarose gel electrophoresis of the PNAC samples depending on the size of the AuNPs in 1× TAE buffer solution. The difference in color (i.e., optical property changes) is due to the size of the AuNPs (20-, 50-, and 100-nm). The experiment was performed twice for each condition.

We performed gel electrophoresis to analyze the uniformity of the PNACs according to the size of the core AuNPs (Supplementary Fig. 4). The narrow and clear band of the 20-nm PNAC indicates their high uniformity, demonstrating that 20-nm PNACs are suitable for our strategy of colorimetric Aβ-degrading drug screening. As the size of the PNACs is increased, their bands are broadened, indicating that the uniformity of larger PNACs is poor. In the case of single AuNPs, the hydrodynamic size of the AuNPs is increased by Aβ aggregation on the AuNP surface, and these larger AuNPs experience more Aβ aggregation, thereby increasing the local Aβ concentration around the AuNPs[1]. The increased local Aβ concentration at the surfaces of the AuNPs may enhance the probability of frequent contact between partially unfolded oligomeric aggregates, resulting in more rapid clustering of the AuNPs and Aβ[2]. This tendency occurs more easily with large-sized AuNPs. The reaction between large-sized AuNPs and Aβ happens rapidly, which interferes with the fabrication of uniform and stable PNACs. For these reasons, the uniformity of PNACs with large-sized core AuNPs is decreased, and their gel electrophoresis bands are broadened.

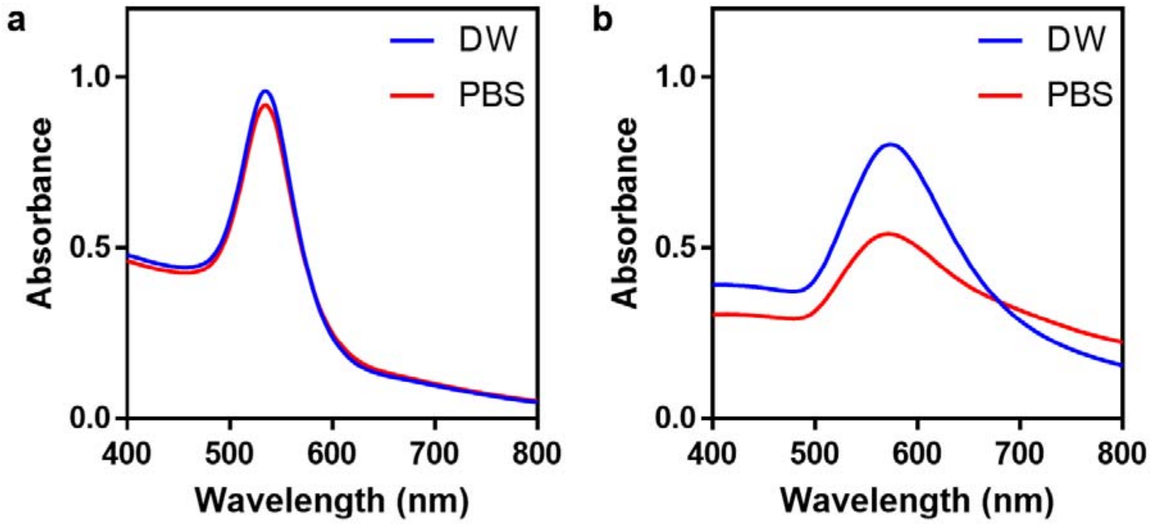

Supplementary Figure 5. a, The shift in the plasmonic peak of the 50-nm PNAC solution, independent of buffer solution composition (distilled water or PBS). **b,** The dependence of the shift in the plasmonic peak of the 100-nm PNAC solution on the buffer solution composition.

We tested the salt resistance of differently sized PNACs in PBS (Supplementary Fig. 5). The results showed that the absorbance peak of 50-nm PNACs is slightly decreased because the 50-nm PNACs become aggregated in PBS. In the case of 100-nm PNACs, their absorbance peak is dramatically decreased. The results indicated that large-sized PNACs became more unstable in salt conditions (PBS) as the PNACs became larger. However, as shown in Supplementary Fig. 8c, the aggregation of 20-nm PNACs is negligible, indicating that the 20-nm PNACs were stable in salt conditions and thus suitable for use in the colorimetric Aβ-degrading drug screening platform.

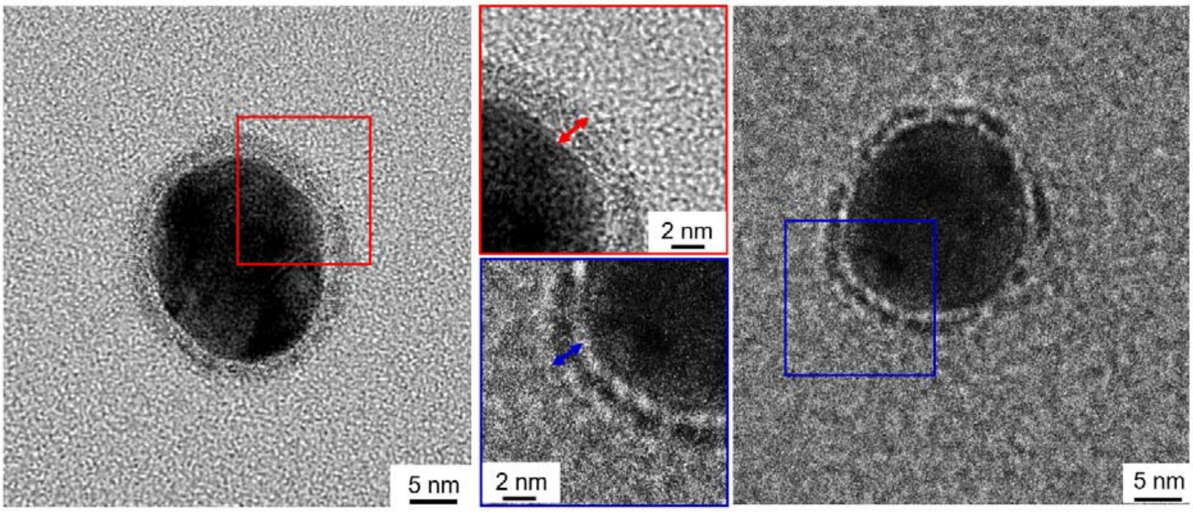

Supplementary Figure 6. The images of a single PNAC using HRTEM (left, red box), and cryo-TEM (right, blue box)

To investigate the formation of the amyloid corona, we compared HRTEM images with the cryo-TEM images (Supplementary Fig. 6). In the case of the HRTEM image, the AuNP is covered with a uniformly thick (~3 nm) amyloid corona layer. The cryo-TEM analysis exhibits that result is similar to the HRTEM image. In detail, the results of cryo-TEM show that the Aβ layer of the PNAC is ~3 nm. Both electron micrographs indicate that the thickness of the Aβ layer of PNAC is similar to that of a single Aβ oligomeric aggregate, as reported previously[3].

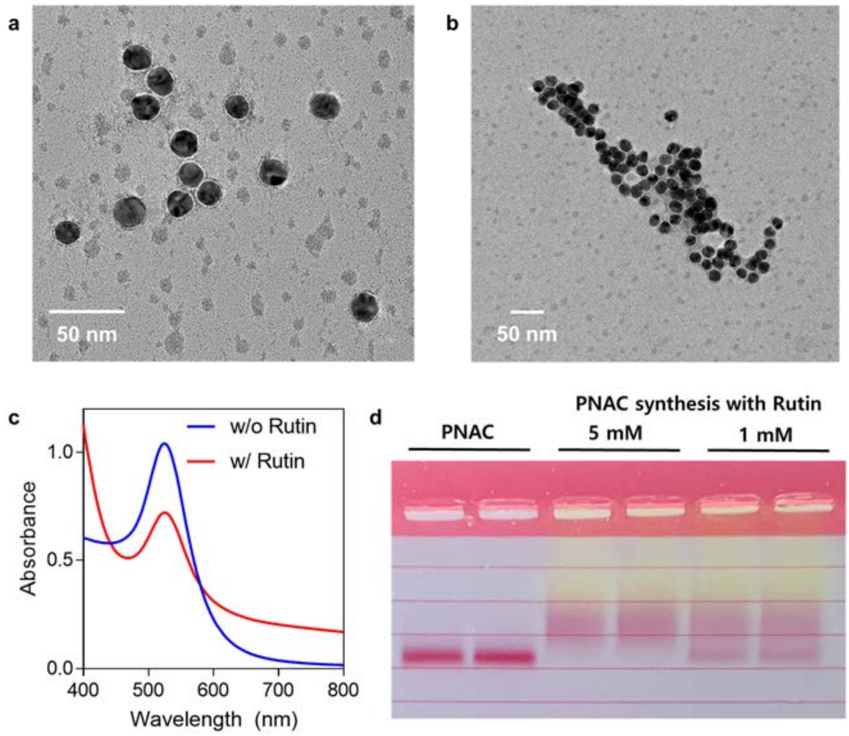

Supplementary Figure 7. Anti-aggregation activity test of PNACs using rutin hydrate. **a**, HRTEM image of PNACs with 5 mM rutin hydrate in DW. **b**, HRTEM image of PNACs with 5 mM rutin hydrate in PBS. **c**, The shift in the plasmonic peak of the PNACs with or without 5 mM rutin hydrate in the PNAC fabrication process. **d**, Agarose gel electrophoresis of PNACs with and without rutin hydrate in the 1× TAE buffer solution.

We added the anti-aggregation agent (i.e., rutin hydrate) during the process of PNAC synthesis. As shown in Supplementary Fig. 7a, the formation of the amyloid corona on AuNPs is restricted compared to that on the PNACs without rutin hydrate (see Fig. 1c). For this reason, the steric stability of PNACs with rutin hydrate is significantly decreased, resulting in aggregation in PBS (Supplementary Fig. 7b). We confirmed the aggregation of PNACs with rutin hydrate by a salt-resistance test (Supplementary Fig. 7c). The PNACs without rutin hydrate are stable in PBS because the amyloid corona on PNAC provides steric stability. In contrast, the PNACs with rutin hydrate are unstable, showing a plasmonic peak shift because rutin hydrate hinders the formation of the amyloid corona of PNAC. We also performed gel electrophoresis as a suitable assay to verify the anti-aggregation effect of rutin hydrate (Supplementary Fig. 7d). The PNACs without rutin hydrate exhibit a distinct band. This is attributed to the good dispersion of the PNACs arising from the good steric stability provided by the amyloid corona. With rutin hydrate during the PNAC fabrication process, the PNAC band is broadened, implying that the aggregation of PNACs occurs because of the low steric stability of PNACs synthesized in the presence of rutin hydrate.

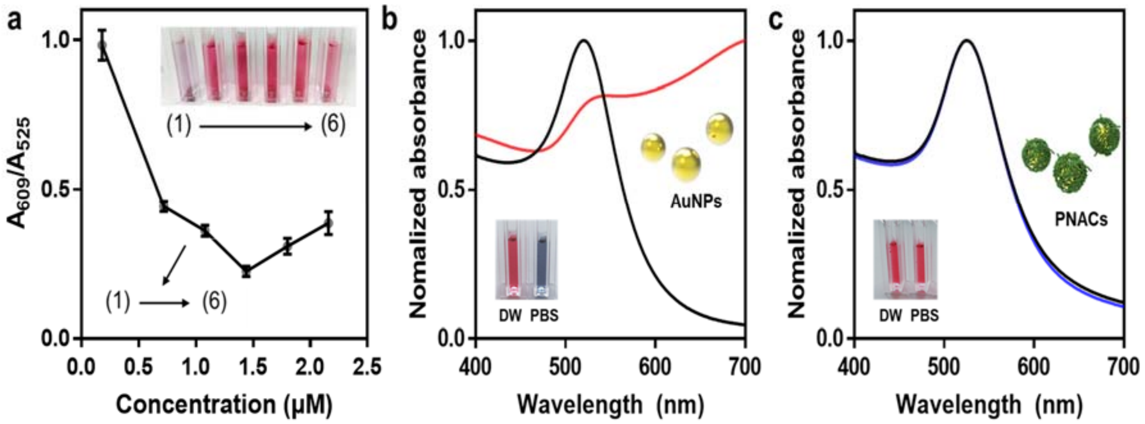

Supplementary Figure 8. a, The A_{609}/A_{525} of the PNAC solution depending on the concentration of Aβ monomers. Inset: Photograph of PNAC solutions containing various concentrations of Aβ monomers: (1) 0.18, (2) 0.72, (3) 1.08, (4) 1.44, (5) 1.8, and (6) 2.16 μM. **b,** The shift in the plasmonic peak of the AuNP solution depending on the type of buffer solution (deionized water or PBS). **c,** The shift in the plasmonic peak of the PNAC solution in DW or PBS. Dot plots in b represent the average ± standard deviation calculated from n = 3 independent samples.

10

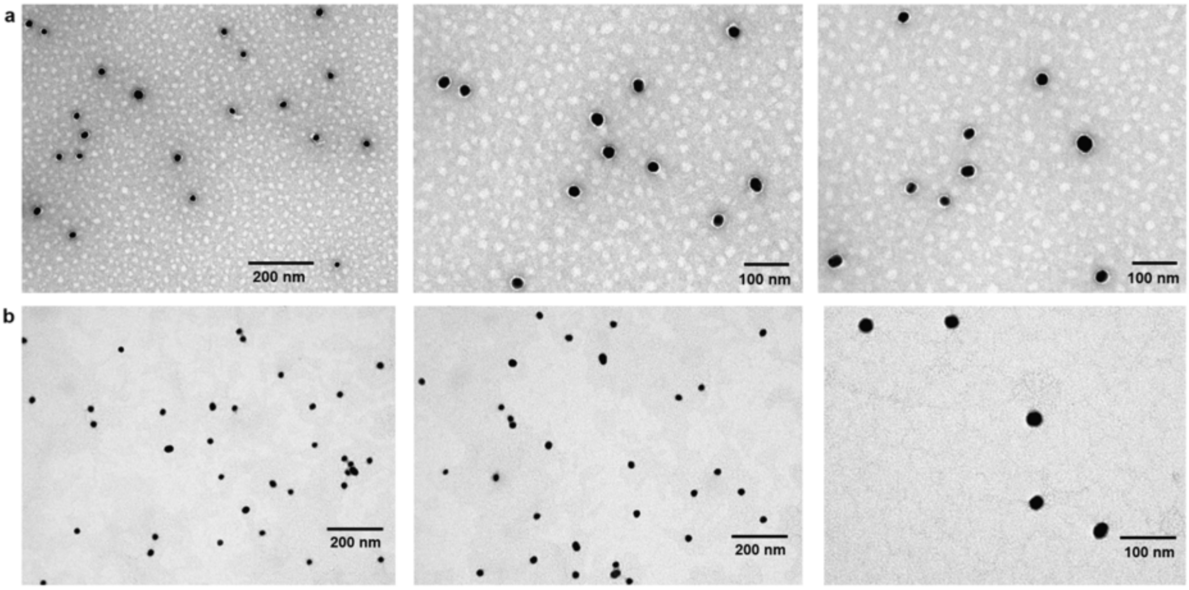

Supplementary Figure 9. High-resolution TEM images of PNACs. **a**, With uranyl acetate staining, **b**, without staining.

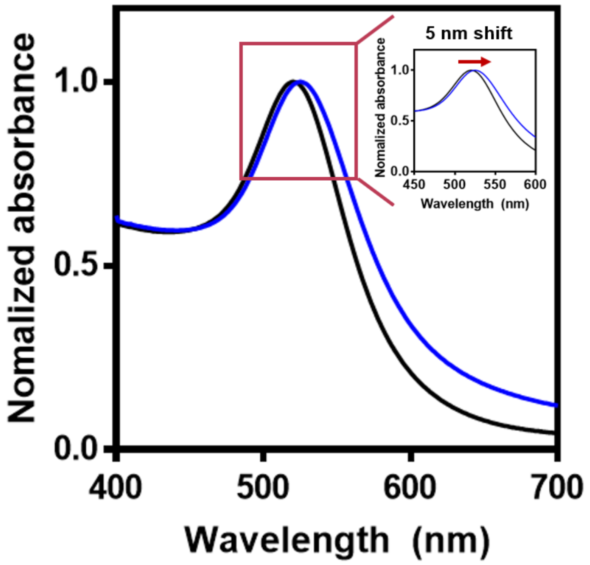

Supplementary Figure 10. Normalized absorbance spectra of the AuNP solution (black line) and PNAC solution (blue line). Inset: Magnification of the LSPR peak shift between the AuNP and PNAC solutions.

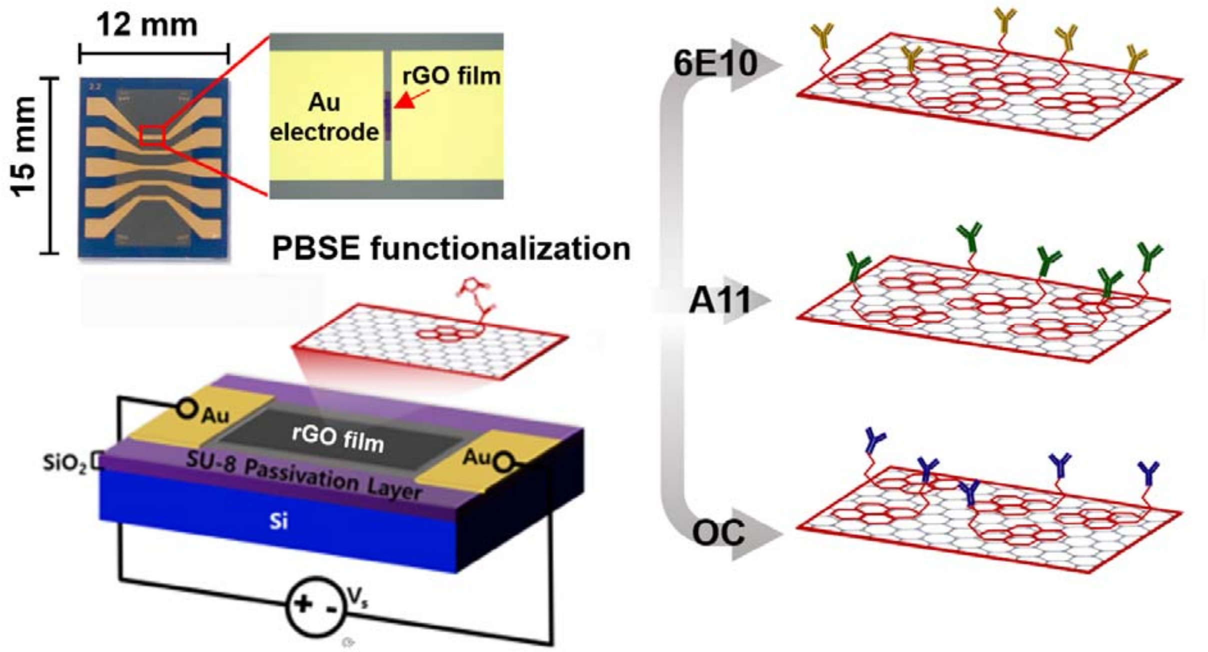

Supplementary Figure 11. Photographic image of the graphene-based sensors and schematic of functionalization of the sensor with three different antibodies (6E10, A11, and OC) and the conformational characterization of the amyloid corona using the graphene-based sensor.

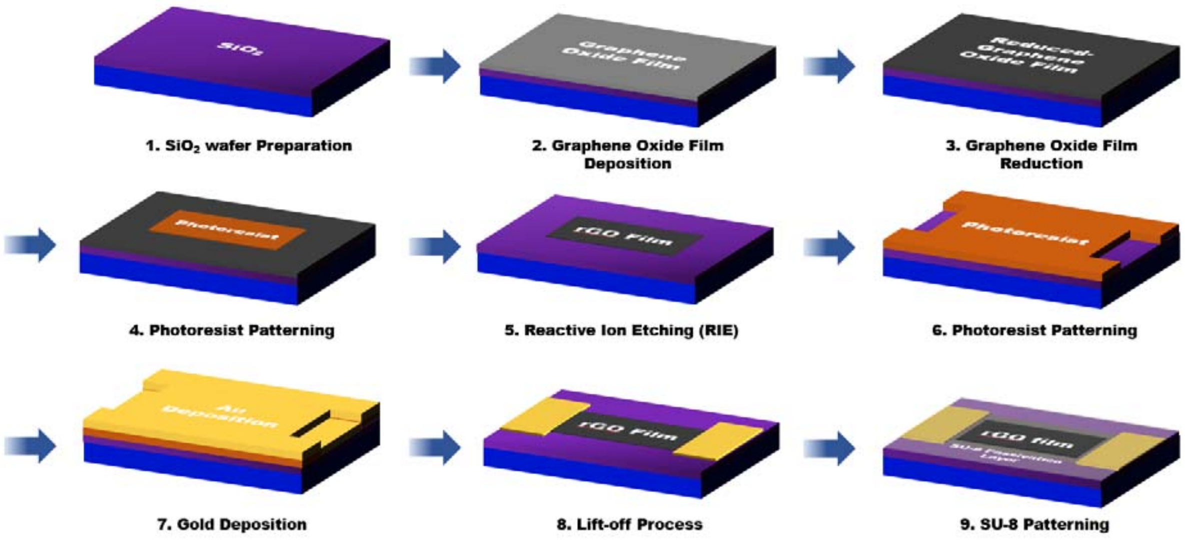

Supplementary Figure 12. The graphene-based sensor fabrication process.

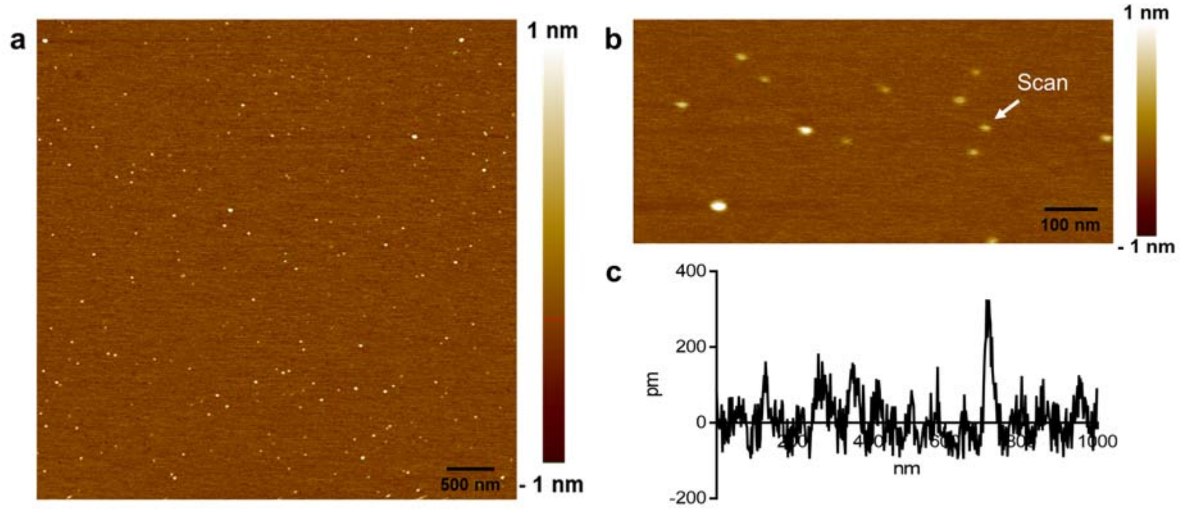

Supplementary Figure 13. AFM characterization of Aβ monomers. **a**, A representative AFM image ($5 \times 5\ \mu m^2$) of the purified Aβ monomers. **b,** A magnified image from a. **c,** The cross-sectional profile of a single Aβ monomer, as indicated by the Scan label, taken from b.

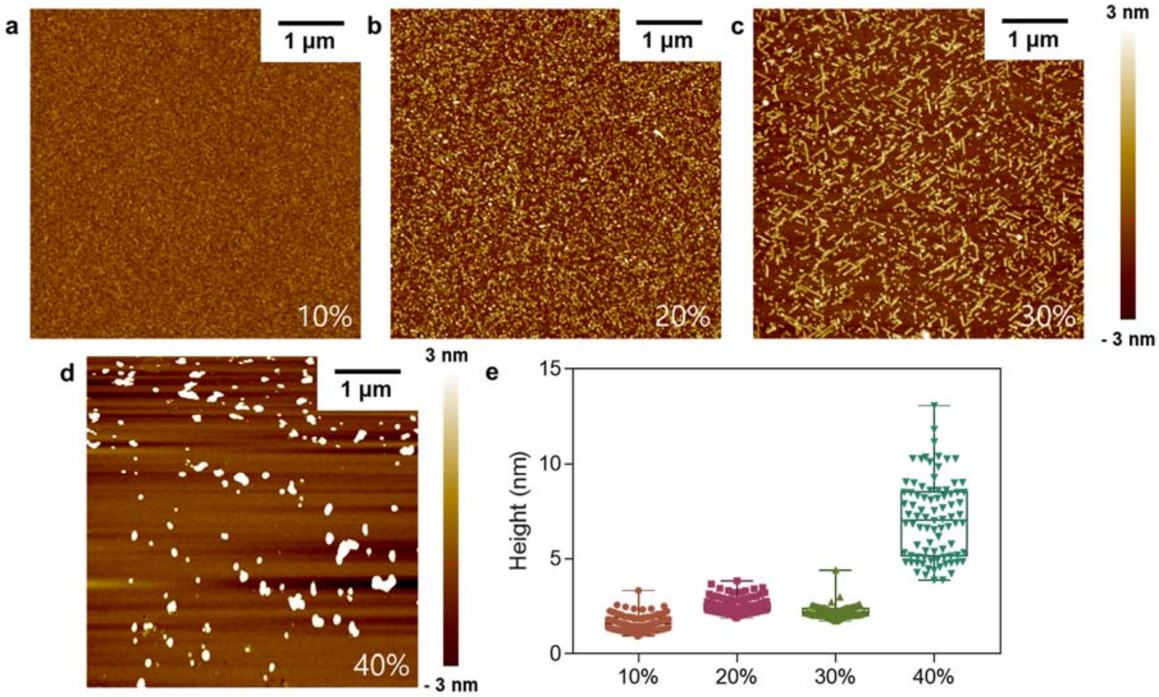

Supplementary Figure 14. AFM characterization of Aβ aggregates present in sucrose fractions. **a**–**d**, Representative AFM images (5 × 5 μm^2) of the Aβ aggregates present in different fractions of the sucrose gradient (10–40%). **e**, Average of Aβ aggregates present in different sucrose fractions. As the concentration of sucrose increases, the mean height of individual aggregates tends to increase. Box plots indicate median (middle line), 25th, 75th percentile (box) and minimum to maximum (whiskers) with showing all points from at least n = 88 independent sample heights.

We prepared Aβ oligomers by incubating the Aβ-monomer solution in PBS (pH 7.4) for 24 h at 4 °C. Then we used the sucrose-gradient centrifugation method[4] to extract purified Aβ oligomers from the incubated Aβ solution (Supplementary Fig. 14a–d).

The results show a significant increase in the average height of the Aβ species as a function of the sucrose fraction density (Supplementary Fig. 14e); a drastic increase in the cross-sectional diameter (i.e., height) of Aβ species is also found in the 40% fraction. The average sizes of Aβ species in each sucrose fraction of 10%, 20%, 30%, and 40% are 1.64, 2.50, 2.23, and 7.28 nm, respectively.

The presence of Aβ oligomers in the 20% sucrose fraction is confirmed by the following:

i) The 30% sucrose fraction (Supplementary Fig. 14c) contains Aβ protofibrils as well as oligomers.

ii) The 2.50-nm average size of Aβ species in the 20% sucrose fraction is similar to that found in the amyloid corona on the PNACs.

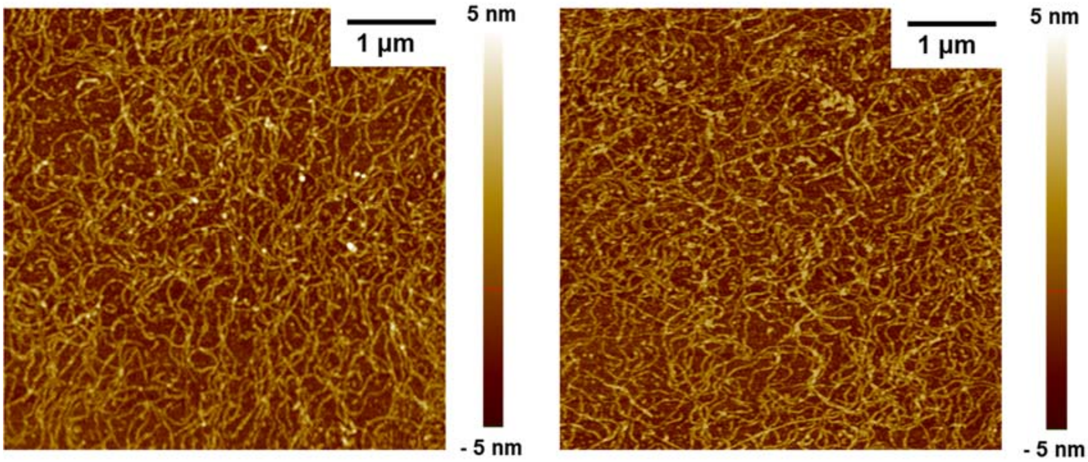

Supplementary Figure 15. AFM images of purified Aβ fibrils (image size: 5 × 5 μm^2).

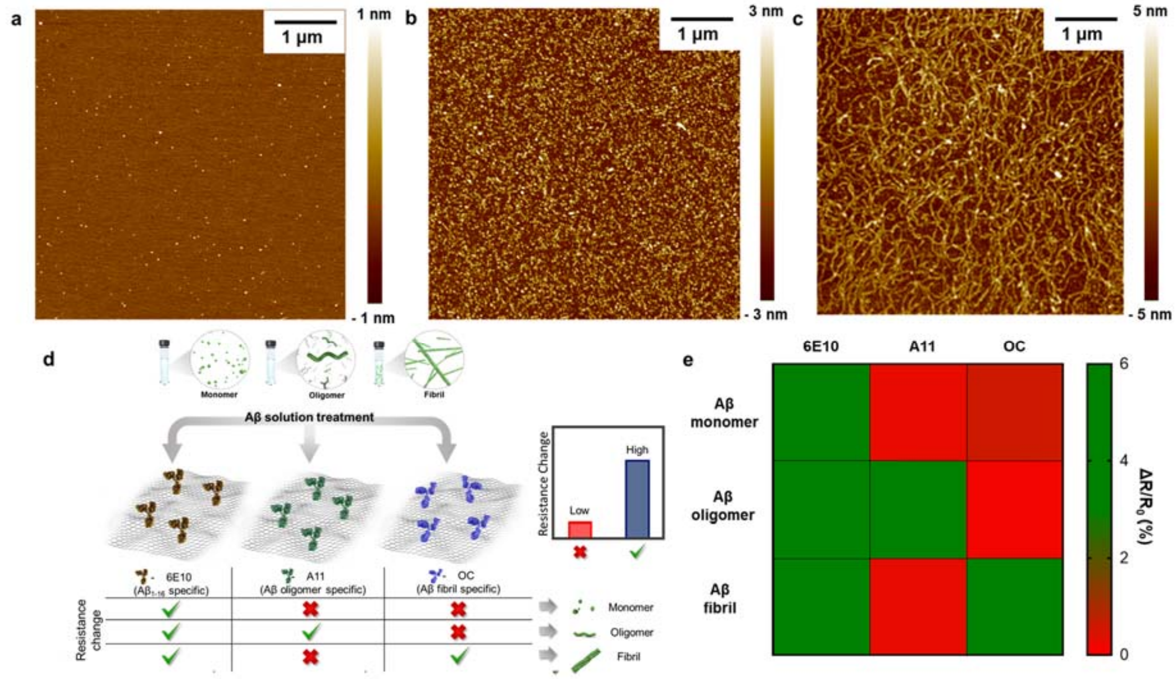

Supplementary Figure 16. Performance test of the graphene-based sensor with three different conformation-specific antibodies. **a**–**c**, Representative AFM images of Aβ monomers, oligomers, and fibrils, respectively. **d**, Schematic of performance test of the graphene-based sensor with the three conformation-specific antibodies 6E10 (brown), A11 (green), and OC (blue). **e**, A heatmap of relative resistance changes of the antibody-immobilized graphene sensor depending on treatment with each purified Aβ solution.

We performed a control experiment to reproduce the table in Fig. 2a using graphene-based sensors wherein each surface of the sensor was functionalized with the different antibodies of monoclonal 6E10, polyclonal A11, and polyclonal OC (Supplementary Fig. 16). We treated the graphene sensors with each purified Aβ solution of monomers, oligomers, and fibrils (Supplementary Fig. 16a–c) and monitored the relative resistance changes of each graphene sensor with the three different types of antibodies (Supplementary Fig. 16d). The results indicate that each graphene sensor shows a specific interaction with each purified Aβ species. The results are expressed as a heatmap (Supplementary Fig. 16e) that is consistent with the table in supplementary Fig. 16d and with Fig. 2a in the manuscript. This control experiment confirms that the conformation present in the amyloid corona on the synthesized PNACs is Aβ oligomeric aggregates.

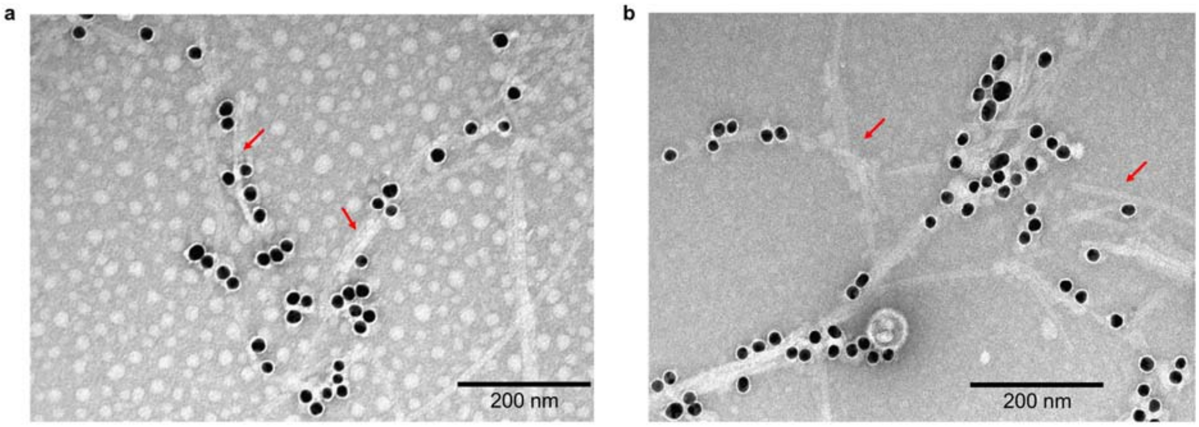

Supplementary Figure 17. TEM images of PNACs attached to Aβ fibril with the addition of 5-μM Aβ monomers (**a**) and 10-μM Aβ monomers (**b**). The red arrows represent Aβ fibrils.

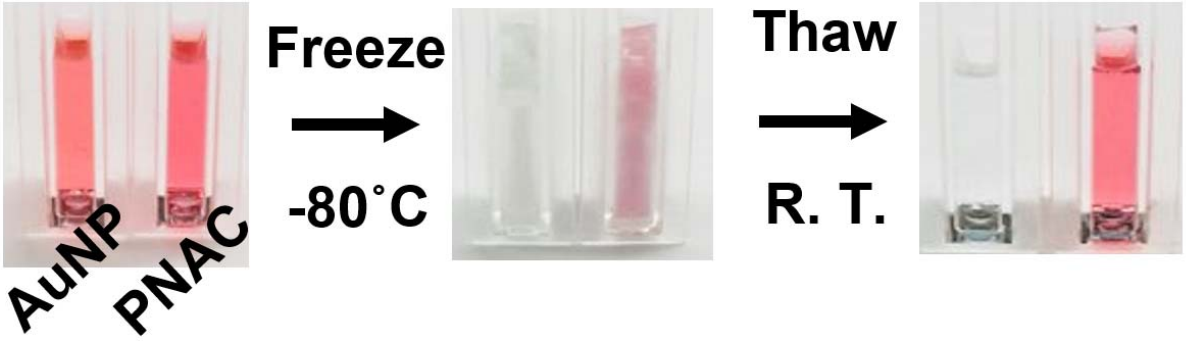

Supplementary Figure 18. Freeze–thaw process of the AuNP and PNAC solutions.

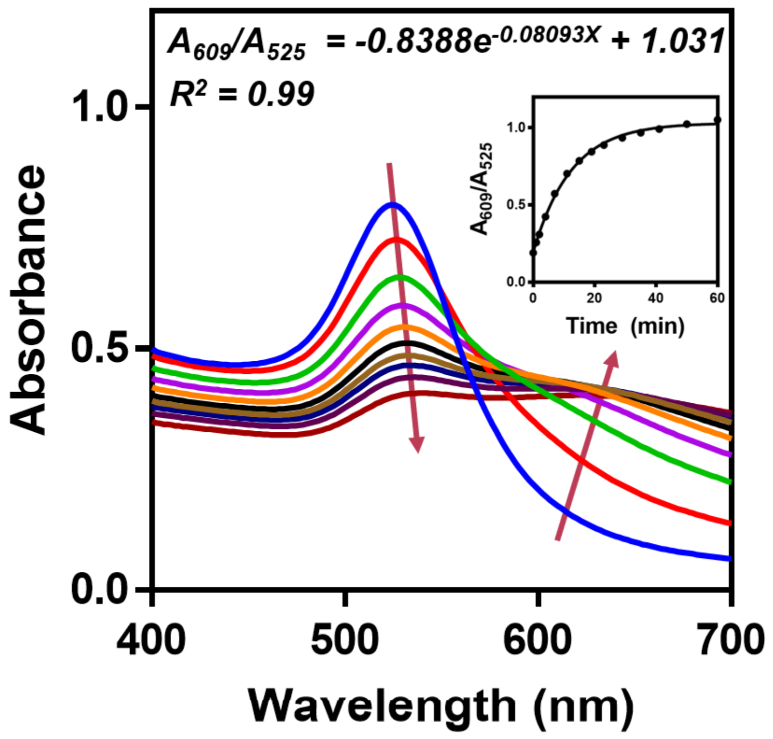

Supplementary Figure 19. The preliminary test of the PNAC-based platform using protease XIV. The A_{609}/A_{525} shift of the PNAC solution was measured for 60 min in the protease XIV solution. Spectra were recorded every 3 min.

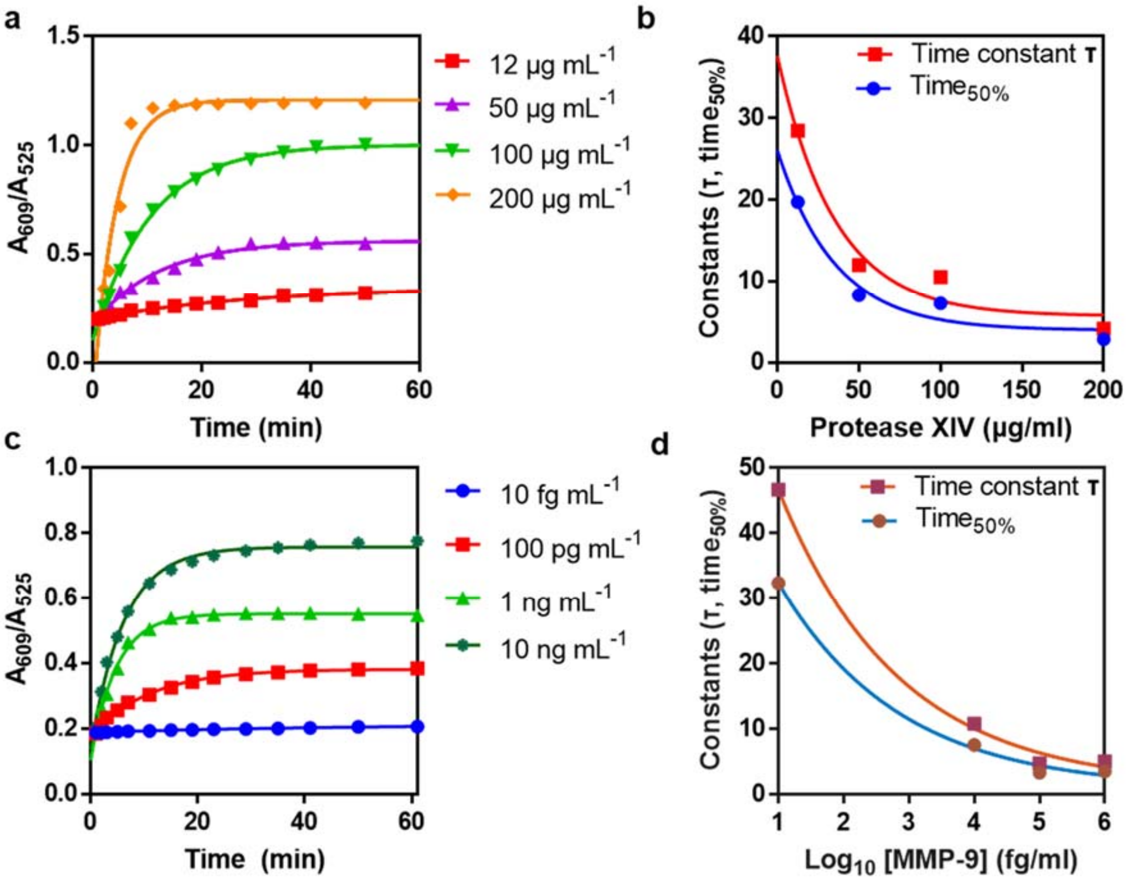

Supplementary Figure 20. Kinetic analyses of Aβ-degrading enzymes protease XIV and MMP-9. **a**, A_{609}/A_{525} of PNAC solutions with various concentrations of protease XIV, measured for 1 h. **b**, The kinetic analysis of Aβ degradation depending on the concentration of protease XIV. **c**, A_{609}/A_{525} of PNAC solutions with various concentrations of MMP-9 measured for 1 h. **d**, Semi-logarithmic plot of the kinetic analysis of Aβ degradation depending on the concentration of MMP-9.

To investigate the time-variable effects of protease XIV, we performed a kinetic analysis of the degradation in the presence of four different concentrations of protease XIV over time for 1 h. As the concentration of protease XIV is increased, the time to reach 50% of the Aβ-degrading drug activity ($Time_{50\%}$) and the time constant (τ) are exponentially decreased (Supplementary Fig. 20b), as shown by Eq. (1) and (2), respectively

$$Time_{50\%} = 22.13 \times e^{-0.02812[\text{protease XIV}]} + 3.939 \quad (1)$$

$$\text{Time constant } (\tau) = 31.94 \times e^{-0.02812[\text{proteaes XIV}]} + 5.684 \quad (2)$$

Moreover, we investigated the time-variable effects of MMP-9 by performing a kinetic analysis of the degradation in the presence of four different MMP-9 concentrations over time for 1 h. As the MMP-9 concentration is increased, $\text{Time}_{50\%}$ and τ are exponentially decreased (Supplementary Fig. 20d), as shown by Eq. (3) and (4), respectively.

$$\text{Time}_{50\%} = 54.24 \times e^{-0.5401[\text{Log MMP}-9]} + 0.7842 \quad (3)$$

$$\text{Time constant } (\tau) = 78.46 \times e^{-0.5453[\text{Log MMP}-9]} + 1.228 \quad (4)$$

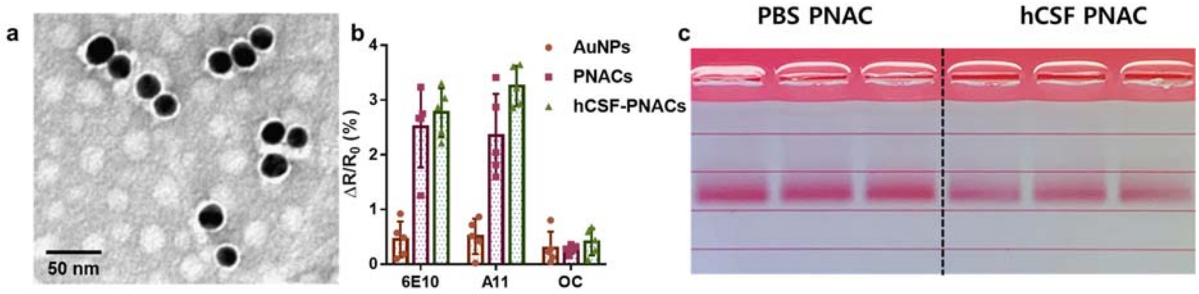

Supplementary Figure 21. hCSF-PNAC characterization. **a**, HRTEM image of hCSF-PNACs. **b**, The relative resistance changes of the antibody-immobilized graphene sensor after treatment with bare AuNPs (orange), PBS-PNACs (wine color), and hCSF-PNACs (green). The bars in b represent the average ± standard deviation calculated from five independent graphene-based sensors. **c**, Agarose gel electrophoresis of the PBS-PNAC and hCSF-PNAC samples in the 1× TAE buffer solution. box plot in b represent the average ± standard deviation calculated from n = 5 independent samples.

To validate corona formation in hCSF-PNACs, we cross-checked the amyloid corona formation of hCSF-PNACs (Supplementary Fig. 21a) and PBS-PNACs by HRTEM (Fig. 1c). In both the images, each AuNP is covered with an amyloid corona of uniform thickness (~3 nm), which corresponds to the size of a single oligomeric aggregate.

To compare the conformational characteristics of hCSF-PNACs and PBS-PNACs, we used graphene sensors wherein each surface of the sensor was functionalized with 6E10, A11, and OC, as in Supplementary Fig. 16. Before the assay using hCSF-PNACs, we confirmed the affinities between the antibodies and bare AuNPs as a negative control. The relative resistance changes, representing the affinities between bare AuNPs and the antibodies, are negligible from all antibodies (Supplementary Fig. 21b). However, with hCSF-PNACs, the relative resistance values of the 6E10- and A11-immobilized sensors are significantly changed by 2.77% and 3.25%, respectively, implying that 6E10 and A11 antibodies have strong affinities with the hCSF-PNACs. In contrast, the relative resistance value of the OC-immobilized sensor remains similar to that of AuNPs, meaning that OC antibodies do not capture hCSF-PNACs. These results indicate that the amyloid corona on hCSF-PNACs comprise not Aβ fibrils, but Aβ oligomeric aggregates. Moreover, we performed gel electrophoresis to analyze the uniformity of hCSF-PNACs compared to that of PBS-PNACs (Supplementary Fig. 21c). The narrow and clear band of hCSF-PNACs is similar to that of PBS-PNACs. This result indicates that the hCSF-PNACs are uniformly fabricated.

This test verified that the conformation of the Aβ in the amyloid corona of hCSF-PNACs was oligomeric aggregates, as in the PBS-PNACs.

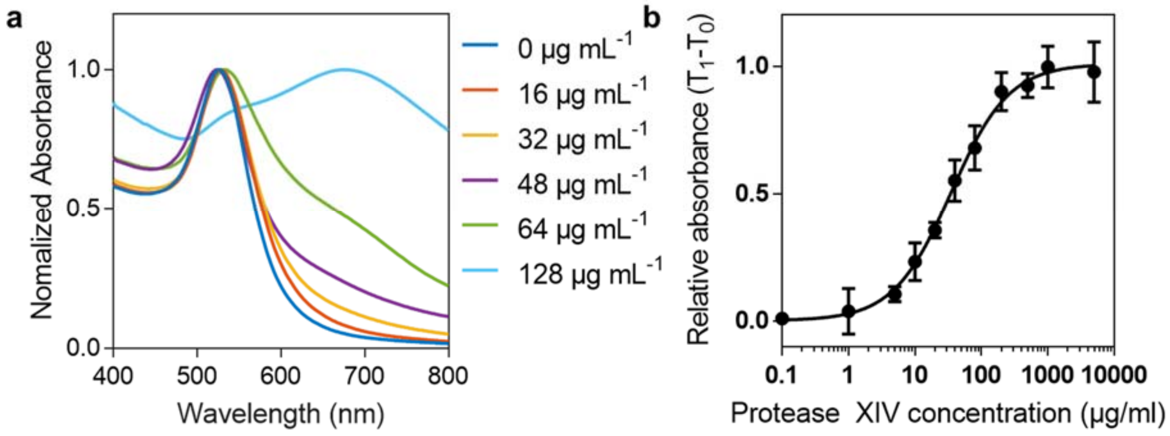

Supplementary Figure 22. **Kinetic analysis of protease XIV using hCSF-PNAC**. **a**, UV-vis spectra of hCSF-PNAC solutions depending on protease XIV concentrations of 0, 16, 32, 48, 64, and 112 μg mL^{-1}. **b**, Plot fitted by the sigmoidal dose-response curve as a function of the protease XIV concentration from 0.1–5000 μg mL^{-1}. Dot plots in b represent the average ± standard deviation calculated from n = 3 independent samples.

We performed a proteolytic activity test using the hCSF-PNACs, as shown in Supplementary Fig. 22, by adding 0, 16, 32, 48, 64, and 112 μg mL^{-1} of protease XIV to the hCSF-PNAC solutions to investigate the Aβ degradation activity. The LSPR peak of hCSF-PNAC is shifted strongly with increases in the protease XIV concentration, which indicates that the amyloid corona of hCSF-PNACs are degraded by the activity of protease XIV (Supplementary Fig. 22a). From the UV-vis spectra, we analyze the change in relative absorbance (T_1−T_0) as a function of the protease XIV concentration using the dose-response model (Supplementary Fig. 22b). We extract the values of the half-maximal effective concentration EC_{50} and the maximal efficacy by the sigmoidal dose-response model (Eq. (5))

$$\Delta \text{ Relative absorbance } (T_1 - T_0) = \frac{1.01}{1+10^{\text{Log}35.19-[\text{Protease XIV}]}},\ R^2 = 0.97 \qquad (5)$$

The values of EC_{50} and maximal efficacy are estimated as 35.19 μg mL^{-1} and 1.01 (a.u.), respectively. These results are consistent with those of the PBS-PNACs.

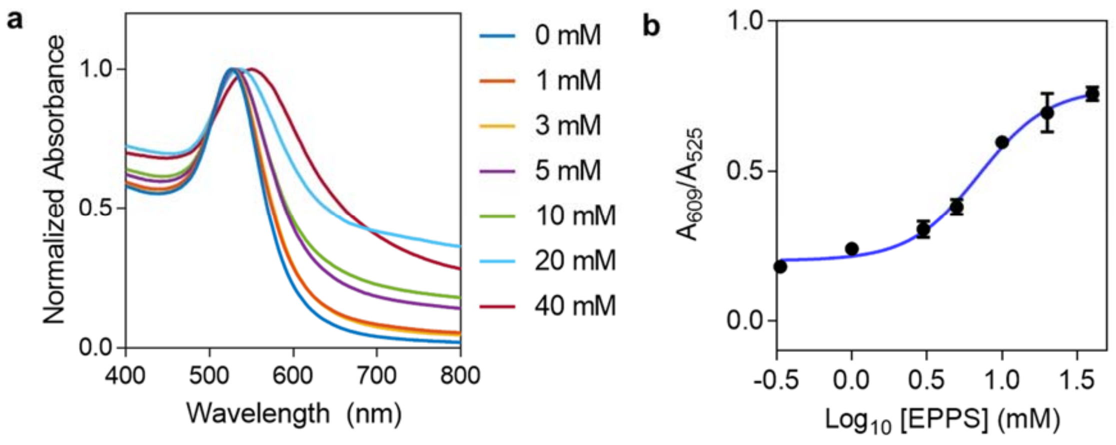

Supplementary Figure 23. **Kinetic analysis of Aβ-degrading agent EPPS using hCSF-PNACs**. **a**, UV-vis spectra of PNAC solution depending on EPPS concentrations of 0, 1, 3, 5, 10, 20, and 40 mM. **b**, A_{609}/A_{525} of PNAC solution depending on EPPS concentration. Dot plots in b represent the average ± standard deviation calculated from n = 3 independent samples.

Beyond studying the activity of Aβ-degrading protease XIV, we measured the efficacy of EPPS, which can degrade all Aβ species (i.e., Aβ oligomers and fibrils) into monomeric Aβ[5], using hCSF-PNACs. In the UV-vis spectra of the hCSF-PNAC solution in the presence of EPPS (Supplementary Fig. 23a), the LSPR peak of the hCSF-PNAC solution is shifted with an increase in EPPS concentration from 1 to 40 mM.

The A_{609}/A_{525} ratio of the hCSF-PNAC solution is logarithmically increased as a function of the EPPS concentration, providing strong evidence of the degradation capacity of EPPS toward the amyloid corona of hCSF-PNACs (Supplementary Fig. 23b). To investigate the pharmacokinetics of EPPS, we extracted the values of EC_{50} and the maximal efficacy by the sigmoidal dose-response model (Eq. (6)).

$$A_{609}/A_{525} = 0.2002 + \frac{0.776}{1+10^{\mathrm{Log}7.038-[\mathrm{EPPS}]}},\ R^2 = 0.98 \qquad (6)$$

The values of EC_{50} and maximal efficacy are estimated as 7.038 mM and 0.776 (a.u.), respectively, consistent with those from PBS-PNAC-based assays (see Fig. 4b).

Altogether, we confirmed that the hCSF-PNACs have the same characteristics as PBS-PNACs; therefore, Aβ-degrading drug screening can be achieved with hCSF-PNAC in a biological environment of hCSF solution.

Supplementary References

1 Kim, Y., Park, J.-H., Lee, H. & Nam, J.-M. How do the size, charge and shape of nanoparticles affect amyloid β aggregation on brain lipid bilayer? Scientific Reports 2016 **6:** 19548.

2 Mahmoudi, M., Kalhor, H. R., Laurent, S. & Lynch, I. Protein fibrillation and nanoparticle interactions: opportunities and challenges. Nanoscale 2013 **5**(7)**:** 2570-2588.

3 Ahmed, M. et al. Structural conversion of neurotoxic amyloid-beta(1–42) oligomers to fibrils. Nature Structural & Molecular Biology 2010 **17**(5)**:** 561-567.

4 De, S. et al. Different soluble aggregates of Aβ42 can give rise to cellular toxicity through different mechanisms. Nature Communications 2019 **10:** 1541.

5 Kim, H. Y. et al. EPPS rescues hippocampus-dependent cognitive deficits in APP/PS1 mice by disaggregation of amyloid-β oligomers and plaques. Nature Communications 2015 **6:** 8997.
