# Supplementary material for: Plasmonic nanoparticle amyloid corona for screening Aβ oligomeric aggregate-degrading drugs
Source: Nat Commun. 2021 Jan 27;12:639. doi: 10.1038/s41467-020-20611-4 (PMC7840768; doi:10.1038/s41467-020-20611-4)
Supplement: Supplementary file 3 — Reporting Summary [file 41467_2020_20611_MOESM3_ESM.pdf]

nature research

Corresponding author(s): Dae Sung Yoon

Last updated by author(s): Nov 2, 2020

Reporting Summary

Nature Research wishes to improve the reproducibility of the work that we publish. This form provides structure for consistency and transparency in reporting. For further information on Nature Research policies, see Authors & Referees and the Editorial Policy Checklist .

Statistics

For all statistical analyses, confirm that the following items are present in the figure legend, table legend, main text, or Methods section.

n/a	Confirmed	
[ ]	[x]	The exact sample size (*n*) for each experimental group/condition, given as a discrete number and unit of measurement
[ ]	[x]	A statement on whether measurements were taken from distinct samples or whether the same sample was measured repeatedly
[ ]	[x]	The statistical test(s) used AND whether they are one- or two-sided<br>*Only common tests should be described solely by name; describe more complex techniques in the Methods section.*
[x]	[ ]	A description of all covariates tested
[x]	[ ]	A description of any assumptions or corrections, such as tests of normality and adjustment for multiple comparisons
[ ]	[x]	A full description of the statistical parameters including central tendency (e.g. means) or other basic estimates (e.g. regression coefficient) AND variation (e.g. standard deviation) or associated estimates of uncertainty (e.g. confidence intervals)
[ ]	[x]	For null hypothesis testing, the test statistic (e.g. *F*, *t*, *r*) with confidence intervals, effect sizes, degrees of freedom and *P* value noted<br>*Give P values as exact values whenever suitable.*
[x]	[ ]	For Bayesian analysis, information on the choice of priors and Markov chain Monte Carlo settings
[x]	[ ]	For hierarchical and complex designs, identification of the appropriate level for tests and full reporting of outcomes
[x]	[ ]	Estimates of effect sizes (e.g. Cohen's *d*, Pearson's *r*), indicating how they were calculated

Our web collection on statistics for biologists contains articles on many of the points above.

Software and code

Policy information about availability of computer code

Data collection	HRTEM imaging:Gatan Microscopy Suite Software®, Galtan DigitalMicrograph® (V 3.21.1374) equipped on a transmission electron microscope (LEO 912AB OMEGA, Germany)<br>Cryo-TEM imaging: Gatan Microscopy Suite Software®, Gatan Digitalmicrograph equipped on a FEI TECNAI F20 coupled with Gatan 4K (2048 x 2048) pixel cooled CCD camera.<br>UV-Vis measurements: UV express® software (V4.1.3) provided by Perkin elmer in Lamda 365.<br>DLS and zeta potential: Zetasizer software 7.03 equipped on NanoZS Malvern instrument.<br>Photographs for recording the color changes of the solutions were taken with an iPhone XR<br>XPS data: Thermo avantage software® (V5.980) provided by Thermo scientific in K-alpha.<br>Resistance measurement: Vlarius V1.6.1 equipped on Keithley 4200A-SCS Semiconductor Parameter Analyzer.<br>AFM imaging: NX-10 (Park Systems, South Korea)<br>Chemical structure: ChemDraw professional (V19.1) provided by PerkinElmer in USA
Data analysis	Drug-response curves (e.g., dose-response curve and time-dependent curve) were fitted by prism7 software (GraphPad)<br>The size of AuNPs was analyzed by imageJ software (V1.8.0_172) (NIH, USA)<br>Significant of results was determined by two-tailed student t-test using prism7 software (GraphPad)<br>AFM data analysis: Smart Scan® (Park Systems, South Korea)

For manuscripts utilizing custom algorithms or software that are central to the research but not yet described in published literature, software must be made available to editors/reviewers. We strongly encourage code deposition in a community repository (e.g. GitHub). See the Nature Research guidelines for submitting code & software for further information.

Data

Policy information about availability of data

All manuscripts must include a data availability statement. This statement should provide the following information, where applicable:

- Accession codes, unique identifiers, or web links for publicly available datasets
- A list of figures that have associated raw data
- A description of any restrictions on data availability

Data supporting the findings of this work are available within the paper and its Supplementary Information file. A reporting summary for this article is available as a Supplementary Information file. The data sets generated and analyzed during the current study are available from the corresponding authors upon reasonable request. The raw data underlying the respective main text (Figs. 1–5) and Supplementary Information (Supplementary Figs. 3, 5, 7, 8, 9, 10, 16, 19, 20, 22, and 23) are provided as a Source Data File.

Field-specific reporting

Please select the one below that is the best fit for your research. If you are not sure, read the appropriate sections before making your selection.

☒ Life sciences ☐ Behavioural & social sciences ☐ Ecological, evolutionary & environmental sciences

For a reference copy of the document with all sections, see nature.com/documents/nr-reporting-summary-flat.pdf

Life sciences study design

All studies must disclose on these points even when the disclosure is negative.

Sample size	Sample sizes were determined based on our and other investigators experiment with graphene-based sensor and coloriemtric drug screening platform. Park et al. [PMID: 32841782], Kim et al. [PMID: 28115249], Choi et al. [PMID: 23777418], Kim et al. [PMID: 28338146], Espargaró et. PMID: 27000658. No statistical method was considered to predetermine sample size, but we observed many statistically significant effects in the data without a prior sample size calculation.
Data exclusions	No data was excluded from analysis.
Replication	All the attempts was repeated independently for at least three times for each sample tested. All replication of experiments was successful.
Randomization	All samples were randomly allocated into each experimental group including colorimetric drug screening test, graphene-based immunoassay, electrophoresis, and etc.
Blinding	All the investigators were blineded to groups allocation during data collection and analysis.

Reporting for specific materials, systems and methods

We require information from authors about some types of materials, experimental systems and methods used in many studies. Here, indicate whether each material, system or method listed is relevant to your study. If you are not sure if a list item applies to your research, read the appropriate section before selecting a response.

Materials & experimental systems

n/a	Involved in the study	
☐	☒	Antibodies
☒	☐	Eukaryotic cell lines
☒	☐	Palaeontology
☒	☐	Animals and other organisms
☒	☐	Human research participants
☒	☐	Clinical data

Methods

n/a	Involved in the study	
☒	☐	ChIP-seq
☒	☐	Flow cytometry
☒	☐	MRI-based neuroimaging

Antibodies

Antibodies used

6E10
Anti-β-Amyloid, 1-16, BioLegend (Cat# 803017) (Previously Covance catalog# SIG-39300).
Anti-β-Amyloid (1-16), Mouse monoclonal, Clone: 6E10, Isotype: IgG1
A11
Anti-β-Amyloid oligomer, Invitrogen (Cat# AHB0052)
Oligomer A11 Polyclonal Antibody, Rabbit Polyclonal, Clone: A11, Isotype: IgG
OC

Anti-Amyloid Fibrils OC Antibody, Rockland Inc. (Cat# 200-401-E87)
Anti-Amyloid Fibrils OC Antibody, Rabbit Polyclonal, Clone: OC, Isotype: IgG

Validation

6E10 was validated in WB, direct ELISA, IHC-P and reported IHC-F, EM in the literature. Cited in 25 publication(s).
A11 was validated in DB, ELISA, IHC, Neu, WB, ICC/IF and IP. Cited in 58 publication(s).
OC was validated in IHC, DB, WB. Cited in 1 publication(s).
